# A fully-automated low-cost cardiac monolayer optical mapping robot

**DOI:** 10.3389/fcvm.2023.1096884

**Published:** 2023-05-22

**Authors:** Peter Lee, Luqia Hou, Faisal J. Alibhai, Rasha Al-attar, Ana Simón-Chica, Andrés Redondo-Rodríguez, Yilin Nie, Maria Mirotsou, Michael A. Laflamme, Gayathri Swaminath, David Filgueiras-Rama

**Affiliations:** ^1^ Novel Arrhythmogenic Mechanisms Program, Centro Nacional de Investigaciones Cardiovasculares Carlos III (CNIC), Madrid, Spain; ^2^ Essel Research and Development Inc., Toronto, ON, Canada; ^3^ Cardiometabolic Department, Merck & Co., Inc., South San Francisco, CA, United States; ^4^ McEwen Stem Cell Institute, University Health Network, Toronto, ON, Canada; ^5^ Peter Munk Cardiac Centre, University Health Network, Toronto, ON, Canada; ^6^ Department of Laboratory Medicine & Pathobiology, University of Toronto, Toronto, ON, Canada; ^7^ Centro de Investigación Biomédica en Red de Enfermedades Cardiovasculares (CIBERCV), Madrid, Spain; ^8^ Instituto de Investigación Sanitaria Hospital Clínico San Carlos (IdISSC), Hospital Clínico San Carlos, Madrid, Spain

**Keywords:** optical mapping, cardiac electrophysiology, automation, robotics, calcium sensitive dyes, voltage sensitive dyes, genetically encoded voltage indicators

## Abstract

Scalable and high-throughput electrophysiological measurement systems are necessary to accelerate the elucidation of cardiac diseases in drug development. Optical mapping is the primary method of simultaneously measuring several key electrophysiological parameters, such as action potentials, intracellular free calcium and conduction velocity, at high spatiotemporal resolution. This tool has been applied to isolated whole-hearts, whole-hearts in-vivo, tissue-slices and cardiac monolayers/tissue-constructs. Although optical mapping of all of these substrates have contributed to our understanding of ion-channels and fibrillation dynamics, cardiac monolayers/tissue-constructs are scalable macroscopic substrates that are particularly amenable to high-throughput interrogation. Here, we describe and validate a scalable and fully-automated monolayer optical mapping robot that requires no human intervention and with reasonable costs. As a proof-of-principle demonstration, we performed parallelized macroscopic optical mapping of calcium dynamics in the well-established neonatal-rat-ventricular-myocyte monolayer plated on standard 35 mm dishes. Given the advancements in regenerative and personalized medicine, we also performed parallelized macroscopic optical mapping of voltage dynamics in human pluripotent stem cell-derived cardiomyocyte monolayers using a genetically encoded voltage indictor and a commonly-used voltage sensitive dye to demonstrate the versatility of our system.

## Introduction

Cardiovascular diseases remain a major cause of death globally. Basic research using optical mapping to measure action potentials, calcium concentrations and conduction velocity at high spatiotemporal resolution has had a major impact on our understanding of such diseases (1–5). And because this tool permits the investigation of cardiac electrophysiology at a macroscopic spatial scale, much has been learned about cardiac excitation wave dynamics during fibrillation. In addition, the use of drugs to perturb normal functioning of ion-channels and gap junctions provides mechanistic insights into pathophysiological behavior (6, 7). However, studies involving the use of existing and new drugs at various concentrations and combinations requires the use of scalable cardiac substrates and scalable measurement technologies. This requirement is further underscored when considering the immense time and money invested in early drug discovery (8, 9).

Currently, optical mapping/imaging is the only multi-parameter measurement technology that is scalable. It has been applied to whole-hearts (*ex vivo* and *in vivo*), tissue-slices and cardiac monolayers/tissue-constructs (1–5). Cardiac monolayers/tissue-constructs (also known as engineered heart tissues) are scalable macroscopic substrates that permit the measurement of several key electrophysiological parameters (10–14). Although many recent advances have been made in optical mapping technologies for these substrates (15–21), there is a scarcity of published work on fully-automated optical mapping systems that not only perform optical mapping, but also perform fluid-handling, electrical/optical stimulation and mechanical positioning, all inside an incubator. To truly increase measurement throughput, all tasks involved in an optical mapping experiment must be automated without human intervention, similar to what has been done in the production and maintenance of human pluripotent stem cell-derived cultures (22, 23) and the analysis of optical mapping data (24).

As a formative project, we aimed at developing a barebones, scalable and fully-automated monolayer optical mapping robot that requires no human intervention, specifically for macroscopic cardiac monolayer substrates large enough to study excitation wave dynamics. High-throughput optical mapping systems for “point” measurements of cardiac cell cultures plated in 96-well plates, for example, have already been developed (16, 25). In effect, each well yields one action potential/calcium transient measurement point (26). Here, we describe and validate the robot using the well-established and inexpensive neonatal-rat-ventricular-myocyte (NRVM) monolayer substrate (27) plated on standard 35 mm dishes by loading cells with dye and treatment and then performing macroscopic optical mapping, all in a parallelized fashion inside a 37°C incubator. To demonstrate future applicability to human pluripotent stem cell-derived cardiomyocyte (hPSC-CM) monolayers/tissue-constructs (28), we performed parallelized macroscopic optical mapping of voltage dynamics in hPSC-CM monolayers also plated on standard 35 mm dishes using a relatively fast genetically encoded voltage indicator (GEVI) and a commonly-used voltage sensitive dye (VSD) (25, 29–31). The total cost of the system components is <$15,000 USD for the oblique excitation configuration and <$20,000 USD for the perpendicular excitation configuration, which requires the use of more components. The total cost of the system is lower than the cost of one optical mapping camera system typically used in the field. By overcoming the limitations of cost and human intervention, we believe the fully-automated high-throughput electrophysiological measurements of macroscopic cardiac constructs can be operated seamlessly.

## Methods

### Optical mapping robot design

**Figure 1:**
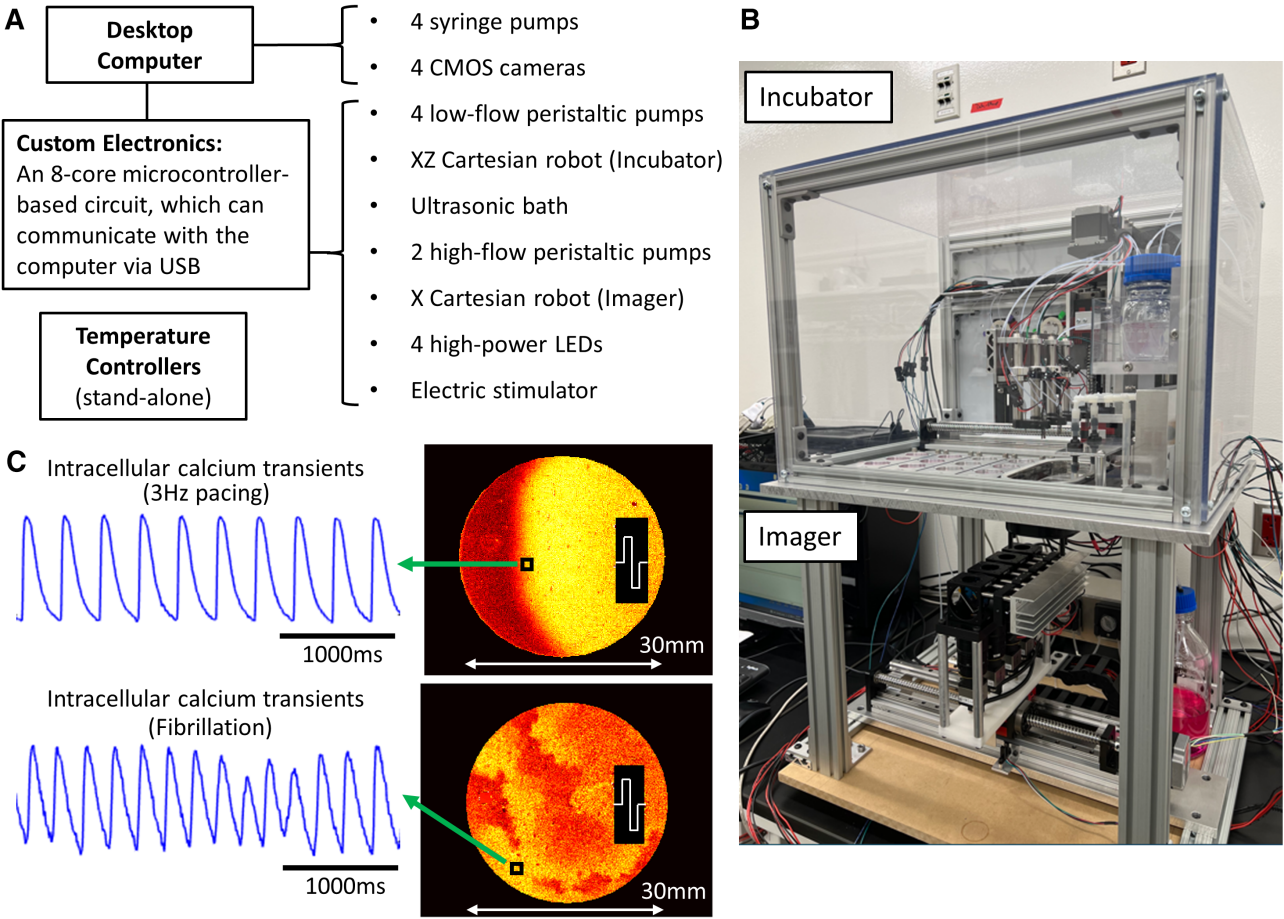
Cardiac monolayer optical mapping robot. (**A**) Major components of the robot and their connection to a standard desktop computer. The temperature controllers operate independently. (**B**) A picture of the optical mapping robot, showing the *Incubator* (top) and *Imager* (bottom). The custom electronics and 2 high-flow peristaltic pumps are not in the field-of-view. (**C**) Sample NRVM calcium data from one of the cameras during 3 Hz electrical pacing and fibrillation. The normalized fluorescence intensity maps at one time point captures the propagation of the activation fronts. The fibrillating monolayer was formed from older neonatal rats (3–4-day-old).

**Figure 2:**
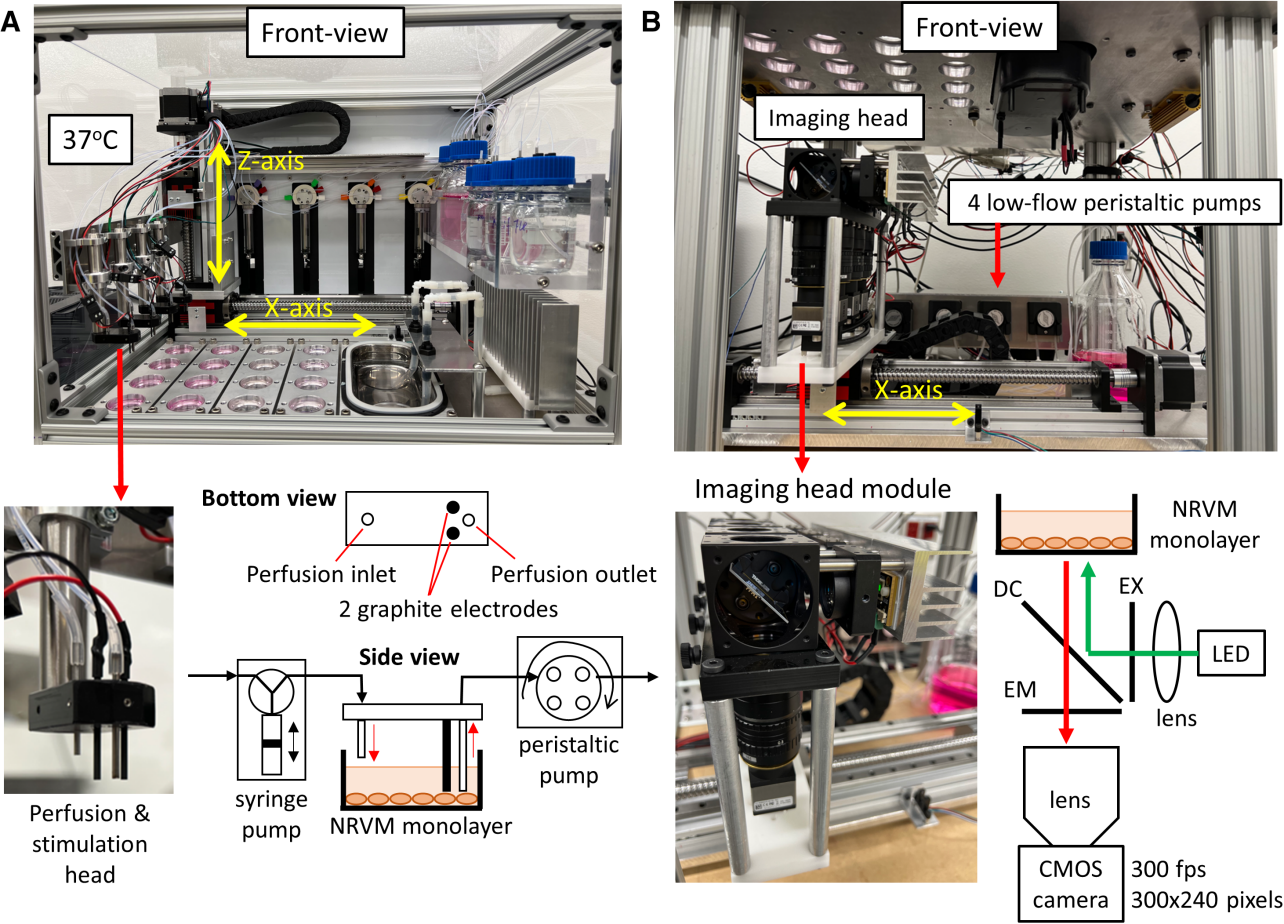
Incubator and imager. (**A**) Front-view picture of the *Incubator* (Supplementary Figure S1A shows the top-view schematic). The 16 monolayers are arranged into 4 columns and a 2-axis XZ Cartesian robot positions the 4 perfusion & stimulation heads into one column of 4 dishes or into the ultrasonic bath, which acts as both a cleaning station and a waste bin. The air heater on the right warms the air to 37°C while the heaters attached to the bottom of the aluminum base-plate warms the plate to 37°C. The 4 multi-port syringe pumps add precise volumes of liquid into the dishes from bottles containing the necessary solutions for the experiment. A close-up view of one of the perfusion and stimulation heads shows the 2 graphite stimulation electrodes and the perfusion inlet and outlet. 4 low-flow peristaltic pumps (Figure 2B) remove solution from dishes via the perfusion outlets. (**B**) Front-view picture of the *Imager* (Supplementary Figure S1B shows the top-view schematic). The imaging head comprises of 4 imaging modules, each capable of imaging one monolayer. The imaging head is mounted on a 1-axis X Cartesian robot that positions the imaging head below one of 4 columns of monolayers. A close-up view of an imaging head module (perpendicular excitation) shows that each module comprises of a high-speed CMOS camera, camera lens, emission filter (EM), dichroic mirror (DC), excitation filter (EX), high-power LED and a collimating lens for the LED light.

The optical mapping robot developed for this study automates all tasks involved in a monolayer/tissue-construct optical mapping experiment. It is referred to as a formative system because it will help pave the way to more complex systems based on specific experimental needs. Figure 1 shows the system and sample NRVM calcium data from one of the four cameras during paced and fibrillatory activity. The top portion of the system is the *Incubator* that maintains the environmental temperature at 37°C (Figure 2A and Supplementary Figure S1A). The aluminum base-plate and the air are temperature controlled with stand-alone feedback controllers. The *Incubator* houses 16 monolayers plated on standard 35 mm dishes, multi-port syringe pumps for adding a precise volume of liquid into the dishes, a 2-axis XZ Cartesian robot for positioning the perfusion and stimulation heads, a refillable ultrasonic bath for cleaning the graphite stimulation electrodes, perfusion inlets and outlets. In addition, all the solutions used were placed in the incubator to keep them warm (Figure 2A). High-flow peristaltic pumps (not shown in the figure) were used to refill the ultrasonic bath and low-flow peristaltic pumps (Figure 2B) were used to remove all the liquid from the 35 mm dishes. Graphite electrodes were used to electrically stimulate a small region of cells on one side of the monolayers (32).

The bottom portion of the system is the *Imager*, which performs parallelized optical mapping with 4 cameras recording simultaneously (Figure 2B and Supplementary Figure S1B). The imaging head is made up of 4 modules, which are in turn made up of a high-speed complementary metal-oxide semiconductor (CMOS) camera, camera lens, emission filter (EM), dichroic mirror (DC), excitation filter (EX), high-power light-emitting-diode (LED) and a collimating lens for the LED light (Figure 2B). The more costly perpendicular excitation configuration is shown in Figure 2B, which was used to optically map calcium dynamics in NRVM monolayers. To reduce the cost of the system, we took advantage of recent advances in low-cost high-speed CMOS cameras (33, 34). The camera used in this study (part #: UI-3060CP-M-GL; IDS Imaging Development Systems GmbH, Obersulm, Germany) was configured to record either 240 × 300 or 180 × 180 superpixels (2 × 2 binning mode; 2 × 2 pixels per superpixel) at 300 frames-per-second (fps) or 400 fps, respectively. A 1-axis X Cartesian robot positions the optical mapping cameras below one of 4 columns of monolayers. To reduce the cost of tests, we used NRVM monolayers for the bulk of the robot development. The inexpensive rhod-2AM calcium dye was used to measure calcium transients and conduction velocity in the NRVM monolayers. Supplementary Movie S1 demonstrates the optical mapping robot in action.

### Optical mapping robot components and cost

A detailed list of the components, part numbers, suppliers and costs can be found in Supplementary Table 1. The total cost of the system components is <$15,000 USD for the oblique excitation configuration and <$20,000 USD for the perpendicular excitation configuration. The cost can be significantly reduced further, for example, by in-house engineering of the perfusion system. For instance, one can build the multi-port syringe pumps instead of purchasing off-the-shelf units.

We used a standard desktop computer running the Windows operating system, but added a 4-port USB3.0 PCI express card (AL00014; IDS Imaging Development Systems GmbH) for camera communication and data acquisition. To communicate with the syringe pumps, a USB to RS232 cable (CHIPI-X10; Future Technology Devices International Ltd., Glasgow, UK) was used in conjunction with a 4-Port USB data hub purchased at a local computer shop.

Authors PL or DF-R can be contacted for further details on components and robot assembly.

### Software, electronics and mechanical engineering

The custom camera and instrumentation software, as well as the custom electronics are described in detail previously, with minor modifications (35). Mechanical design and machining of parts were performed in-house using stock aluminum, polycarbonate plastic and acetal plastic. Fastening and joining hardware were purchased either at local hardware shops or McMaster-Carr Supply Company (Elmhurst, IL, USA).

Authors PL or DF-R can be contacted for further details on the software, electronics and mechanical engineering.

### Isolation and culture of NRVM monolayers, dye loading and drug testing

All procedures were approved by the Institutional Ethics Committee for Use and Care of Laboratory Animals at Merck & Co., Inc., Kenilworth, NJ, USA. Timed pregnant *Sprague-Dawley rats* were obtained from a vendor (Charles River Laboratories Inc., Wilmington, MA, USA), housed individually, and monitored until pups were given birth. The isolation and culture techniques are described previously (36). Briefly, 1–2-day-old neonatal rats were placed on ice with paper diapers to achieve hypothermia for anesthesia purpose. Decapitation was used to euthanize the rats. Hearts were removed and collected in D1 solution (Neomyt Kit; Cellutron Life Technologies, Baltimore, MD, USA). Ventricles were then isolated and digested in D2 solution (Neomyt Kit; Cellutron Life Technologies, Baltimore, MD). Two 45-minute pre-plating periods were used to avoid noncardiomyocyte attachment. NRVM were cultured in NS medium (Cellutron Life Technologies, Baltimore, MD) with 100 µM bromodeoxyuridine (Sigma-Aldrich, Burlington, MA, USA). Finally, cardiomyocytes were plated in 35 mm tissue culture dishes coated with SureCoat (Cellutron Life Technologies, Baltimore, MD) at a density of 1.5 × 10^6^ cells/dish for monolayers. NRVM monolayers were cultured at 37°C, 5% CO_2_ for 3–4 days before optical mapping experiments.

NRVM monolayers were stained in Hanks’ balanced salt solution (HBSS) (Waltham, MA) with 5 µM rhod-2AM (Biotium Inc., Hayward, CA, USA) at 37°C for 45 minutes. For the drug effect experiments, monolayers were immersed in HBSS containing the drug under investigation (3 µM nifedipine, N7634/0.6 µM flecainide, F6777; Sigma-Aldrich, Burlington, MA). The vehicle was 0.1% DMSO. Monolayers were incubated with treatment at 37°C for 15 minutes.

### Generation of human embryonic stem cell (hESC)-derived cardiomyocytes and Dye loading

Wild type (WT) and accelerated sensor of action potential 1 (ASAP1)-expressing ESI-17 hESCs (Lineage Cell Therapeutics Inc., Carlsbad, CA, USA) were expanded using mTeSR1 medium (STEMCELL Technologies Inc., Vancouver, BC, Canada) and differentiated into cardiomyocytes via a previously reported growth factor-based guided differentiation protocol (31). Generated hESC-derived cardiomyocytes had a mean cardiac purity of 94.4% ± 1.8% based on flow cytometry for the cardiomyocyte marker cardiac troponin T (Clone: REA400; Miltenyi Biotec North America, Waltham, MA, USA). All hESC experiments were conducted with the approval from the Canadian Institutes of Health Research (CIHR) Stem Cell Oversight Committee (SCOC).

For acquisition of optical action potentials, WT and ASAP1^+^ hESC-CMs were seeded in a 35 mm tissue culture dish coated with growth factor-reduced Matrigel (Corning; Sigma-Aldrich Canada Co., Oakville, ON, Canada) at 1.2 × 10^5^ cells/cm^2^ and maintained with RPMI media supplemented with L-glutamine (2 mM) and insulin containing B27 (Thermo Fisher Scientific Inc., Waltham, MA, USA). Four to six days after seeding cells, optical action potentials were assessed using either the genetically encoded fluorescent voltage sensor ASAP1 (29, 31) or the synthetic VSD FluoVolt (Thermo Fisher Scientific Inc.). For the latter, wild type cells were loaded with FluoVolt for 30 minutes at 37°C. Cells were imaged under both spontaneous and paced conditions while bathed in a modified Tyrode's solution containing (in mM) 140 NaCl, 5.4 KCl, 1 MgCl_2_, 1.8 CaCl_2_, 0.33 NaH_2_PO_4_, 5 glucose, and 10 HEPES, adjusted to pH 7.4 with NaOH.

## Results

Here we present data from a sample 16-monolayer experimental run. The robot tasks were pipelined because of the dye and treatment incubation times and the time needed to perform fluid-handing and optical mapping tasks (Supplementary Figure S2). For drugs, we used (1) nifedipine, a calcium-channel inhibitor that reduces the calcium transient amplitude (37) and (2) flecainide, a sodium-channel inhibitor that reduces conduction velocity (38). The vehicle for both drugs was DMSO. In this sample run, 5 out of 16 NRVM monolayers entered spontaneous fibrillatory activity, which is a well-known disadvantage of using NRVM monolayers plated on 35 mm dishes. However, this disadvantage is tolerated because of the simplicity and low-cost of this preparation.

**Figure 3:**
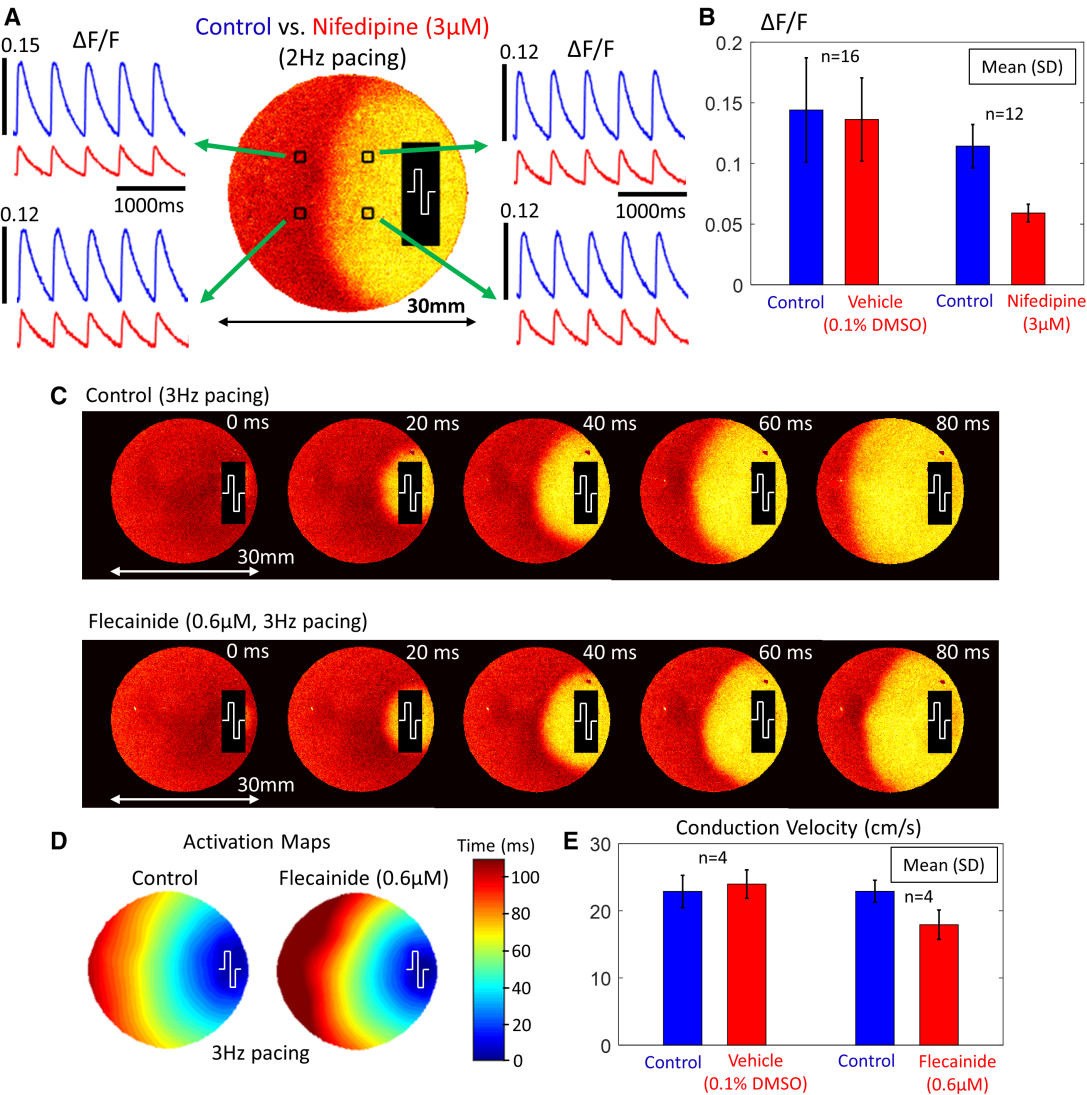
Calcium transient amplitude and conduction velocity changes (NRVM monolayers). (**A**) Sample calcium transients from 4 points in the monolayer during 2 Hz electrical pacing. The stimulation site is shown at the right side of the dish. (**B**) A comparison of the calcium transient amplitude changes between vehicle (0.1% DMSO) and nifedipine (3 µM) treatment. There was a decrease in amplitude following nifedipine treatment. (**C**) Normalized fluorescence intensity maps at progressive time points during 3 Hz electrical pacing before and after flecainide treatment. The stimulation site is shown at the right side of the dish. (**D**) Activation maps before and after flecainide treatment. (**E**) A comparison of the conduction velocity changes between vehicle (0.1% DMSO) and flecainide (0.6 µM) treatment. There was a decrease in conduction velocity following flecainide treatment.

The 11 non-fibrillating monolayers were divided into 3 treatment categories: vehicle (0.1% DMSO, 4 monolayers), nifedipine (3 µM, 3 monolayers) and flecainide (0.6 µM, 4 monolayers). Figures 3A,B shows sample results comparing the effects of nifedipine and vehicle treatments on the calcium transient amplitude during 2 Hz pacing. The calcium transient amplitude was measured right before (control) and then 15 minutes after treatment using ΔF/F as a measure. The sample traces in Figure 3A show a decrease in amplitude after nifedipine treatment. The value of ΔF/F went from 0.1143 ± 0.0178 (control) to 0.0591 ± 0.0073 after nifedipine treatment (*n* = 12; 3 dishes and 4 points per dish) whereas ΔF/F went from 0.1441 ± 0.0430 (control) to 0.1363 ± 0.0343 after vehicle treatment (*n* = 16; 4 dishes and 4 points per dish). Figure 3B shows the decrease in calcium transient amplitude after nifedipine treatment compared to vehicle treatment.

Figures 3C–E shows sample results comparing the effects of flecainide and vehicle treatments on the conduction velocity during 3 Hz pacing. The conduction velocity was measured right before (control) and then 15 minutes after treatment along the diameter of the dish. We used the time fluorescence rises to 50% of its peak change as the “activation time” for a propagating wavefront. Figure 3C shows normalized fluorescence intensity maps at progressive time points during 3 Hz electrical pacing and Figure 3D shows the corresponding activation maps. It can be seen that flecainide slows the propagation speed of the activation front. The conduction velocity went from 22.9 cm/s ± 1.6 cm/s (control) to 17.9 cm/s ± 2.2 cm/s after flecainide treatment (*n* = 4; 4 dishes) whereas conduction velocity went from 22.9 cm/s ± 2.4 cm/s (control) to 24.0 cm/s ± 2.1 cm/s after vehicle treatment (*n* = 4; 4 dishes). Figure 3E shows the decrease in conduction velocity after flecainide treatment compared to vehicle treatment. Supplementary Movie S2 shows parallelized optical mapping with 4 cameras recording simultaneously under one column of NRVM monolayers during a control measurement.

**Figure 4:**
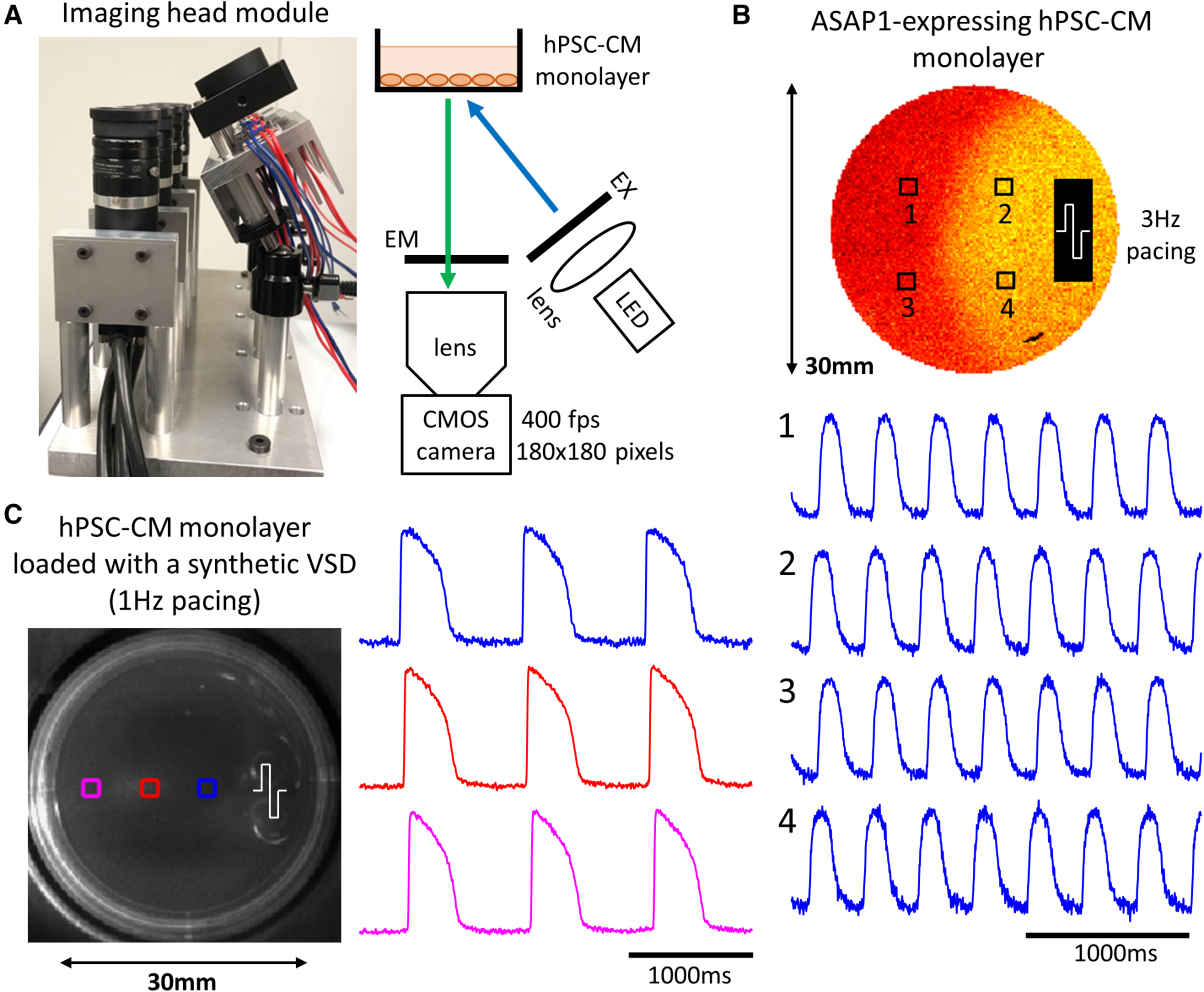
Imaging action potentials using a GEVI and a synthetic VSD in hPSC-CM monolayers. (**A**) A close-up view of a lower-cost imaging head module (oblique excitation) shows that each module comprises of a high-speed CMOS camera, camera lens, emission filter (EM), excitation filter (EX), high-power LED and a collimating lens for the LED light. (**B**) Sample action potentials (normalized signals) from 4 points in the monolayer during 3 Hz electrical pacing. The stimulation site is shown at the right side of the dish. GEVIs have slower kinetics compared to synthetic VSDs. (**C**) Sample action potentials (normalized signals) from 3 points in the monolayer during 1 Hz electrical pacing. The stimulation site is shown at the right side of the dish.

As a proof-of-principle experiment for future development, we performed parallelized macroscopic optical mapping of hPSC-CM monolayers plated on 35 mm dishes using the GEVI ASAP1 (*n* = 4) and the synthetic VSD FluoVolt (*n* = 8). Figure 4A shows the lower-cost imaging head used (oblique excitation configuration). This imaging head is made up of 4 modules, which are in turn made up of a high-speed CMOS camera, camera lens, emission filter (EM), excitation filter (EX), high-power LED and a collimating lens for the LED light. Figure 4B shows sample action potentials from an ASAP1-expressing hPSC-CM monolayer paced at 3 Hz (Supplementary Movie S3). For a higher ΔF/F and faster kinetics, we loaded wild type hPSC-CM monolayers with a commonly-used synthetic VSD FluoVolt. Figure 4C shows sample action potentials during 1 Hz paced activity (Supplementary Movie S4).

## Discussion

To accelerate the elucidation of disease mechanisms and development of effective non-proarrhythmic drugs, high-throughput and low-cost electrophysiological measurement systems must be developed. This necessitates a scalable cardiac substrate and a scalable measurement technology. Here, we described and validated a robot using the inexpensive NRVM monolayer substrate and the clinically-relevant hPSC-CM monolayer substrate, plated on standard 35 mm dishes, by automating all tasks involved in a basic optical mapping experiment. With the total cost of the system components being <$15,000 USD for the oblique excitation configuration, we believe that fully-automated high-throughput electrophysiological measurements of macroscopic cardiac constructs will become more widespread and will enable the investigation of many variations and combinations of drug perturbations in a significantly reduced time frame.

The cost difference between the two imaging head options was significant and the oblique excitation configuration is recommended in cases where there are no geometric constraints. Perpendicular excitation is a more widely-used configuration in fluorescence imaging and combines excitation and emission light into a single optical path. Such a configuration is advantageous in cases where there are geometric constraints on oblique excitation but disadvantageous in certain multi-parametric imaging scenarios where the excitation wavelength of a fluorophore is longer than the emission of another (39–41). The oblique excitation configuration avoids spectral congestion and more easily takes advantage of multi-band emission optical filter technology but has more geometric constraints because the excitation light must illuminate the substrate at an angle.

To more thoroughly study cardiac electrophysiology mechanisms, longitudinal measurements are needed. Such measurements will require the integration of an off-the-shelf or custom 5% CO_2_ incubator, which has become a more affordable option with the development and availability of optical gas sensors (42, 43). And with the addition of microscopy, we believe that the next generation of optical mapping robots will yield new observations important to both the drug-development and basic science research communities.

## Limitations

Our barebones system has limitations and can certainly be improved upon based on experimental needs. Other parameters, such as force/contractility can be measured acutely, or longitudinally over several days and even weeks. As noted earlier, longitudinal measurements will require the integration of an incubator that maintains a 5% CO_2_ environment and a method for maintaining high humidity over cell cultures while not damaging surrounding electrical, mechanical and optical instrumentation. With the emergence of relatively affordable commercial sources of human induced pluripotent stem-cell-derived cardiomyocytes (iPSC-CMs) (17) and the large-scale production of mature hPSC-CMs (31), NRVM monolayers are largely being replaced as a standard experimental cell culture model (10, 14). Any future development of high-throughput systems should focus on the use of human stem-cell-derived cardiomyocytes. The use of brighter GEVIs, like ASAP3 (44), and genetically encoded calcium indicators, like GCaMP (45, 46), can also simplify longitudinal measurements by eliminating the need for repeated dye loading.

To reduce the incidence of spontaneous fibrillatory activity (Figure 1C), we also investigated the use of small-width rectangular monolayers by gluing custom-made polydimethylsiloxane (PDMS) channels to off-the-shelf tissue-culture-treated polystyrene slides and found the incidence of fibrillation drastically reduced (Supplementary Figure S3). Not only does this easily-implemented geometry reduce the number of cells needed, but it simplifies conduction velocity measurements and more readily permits the imaging of multiple monolayers from a single camera sensor (Supplementary Movie S5). Lastly, if one has the capacity to generate hundreds or thousands of hPSC-CM monolayers/tissue-constructs at a macroscopic scale, a more advanced robot should be able to handle trays of cell culture dishes to enable higher-throughput optical mapping experiments.

## Data Availability

The raw data supporting the conclusions of this article will be made available by the authors, without undue reservation.
