# Supplementary material for: A fully-automated low-cost cardiac monolayer optical mapping robot
Source: Front Cardiovasc Med. 2023 May 22;10:1096884. doi: 10.3389/fcvm.2023.1096884 (PMC10240081; doi:10.3389/fcvm.2023.1096884)
Supplement: Supplementary file 1 [file Datasheet1.pdf]

SUPPLEMENTARY MATERIAL

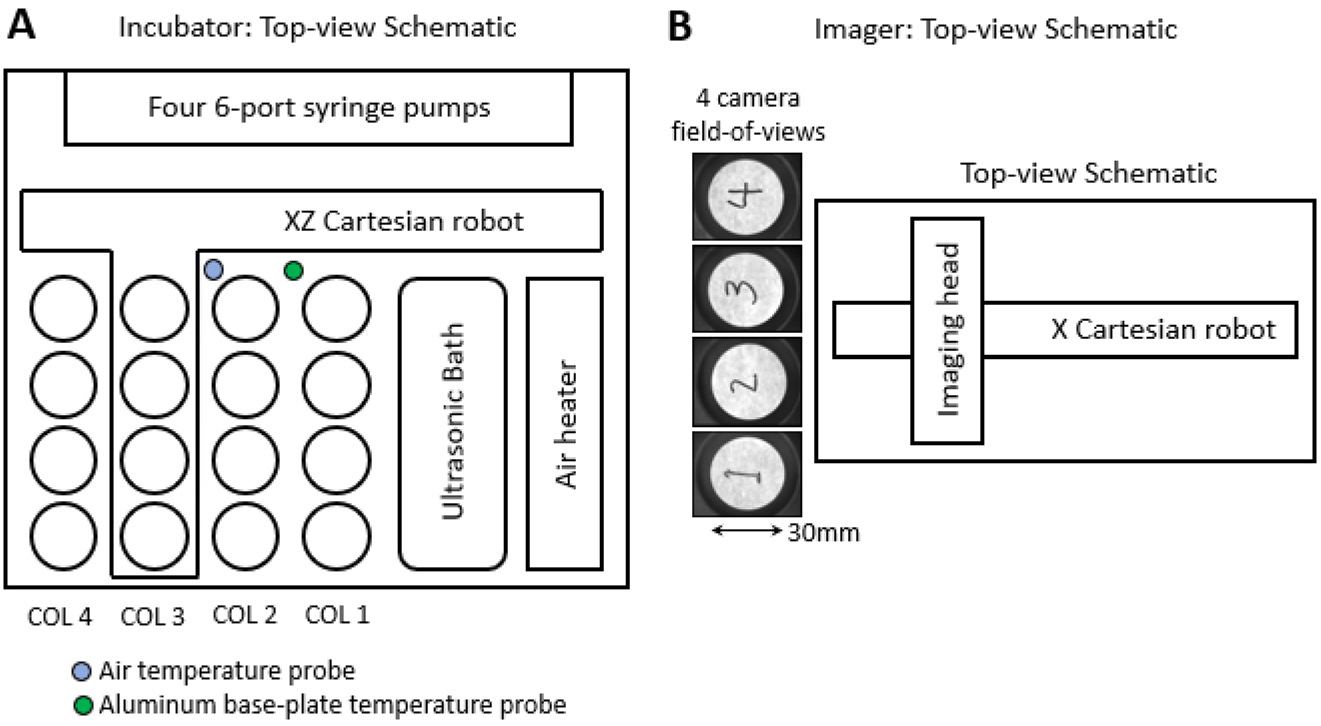

Suppl. Figure 1. Incubator and Imager schematics.
(A) Top-view schematic of the *Incubator*. The 16 monolayers are arranged into 4 columns and a 2-axis XZ Cartesian robot positions the 4 perfusion & stimulation heads into one column of 4 dishes or into the ultrasonic bath, which acts as both a cleaning station and a waste bin. The air heater on the right warms the air to 37°C while the heaters attached to the bottom of the aluminum base-plate warms the plate to 37°C. The 4 multi-port syringe pumps add precise volumes of liquid into the dishes from bottles containing the necessary solutions for the experiment.
(B) Top-view schematic of the *Imager*. The imaging head comprises of 4 imaging modules, each capable of imaging one monolayer. The imaging head is mounted on a 1-axis X Cartesian robot that positions the imaging head below one of 4 columns of monolayers. 4 camera field-of-views show images of numbers written with black marker on the inside bottom-surface of 35mm dishes.

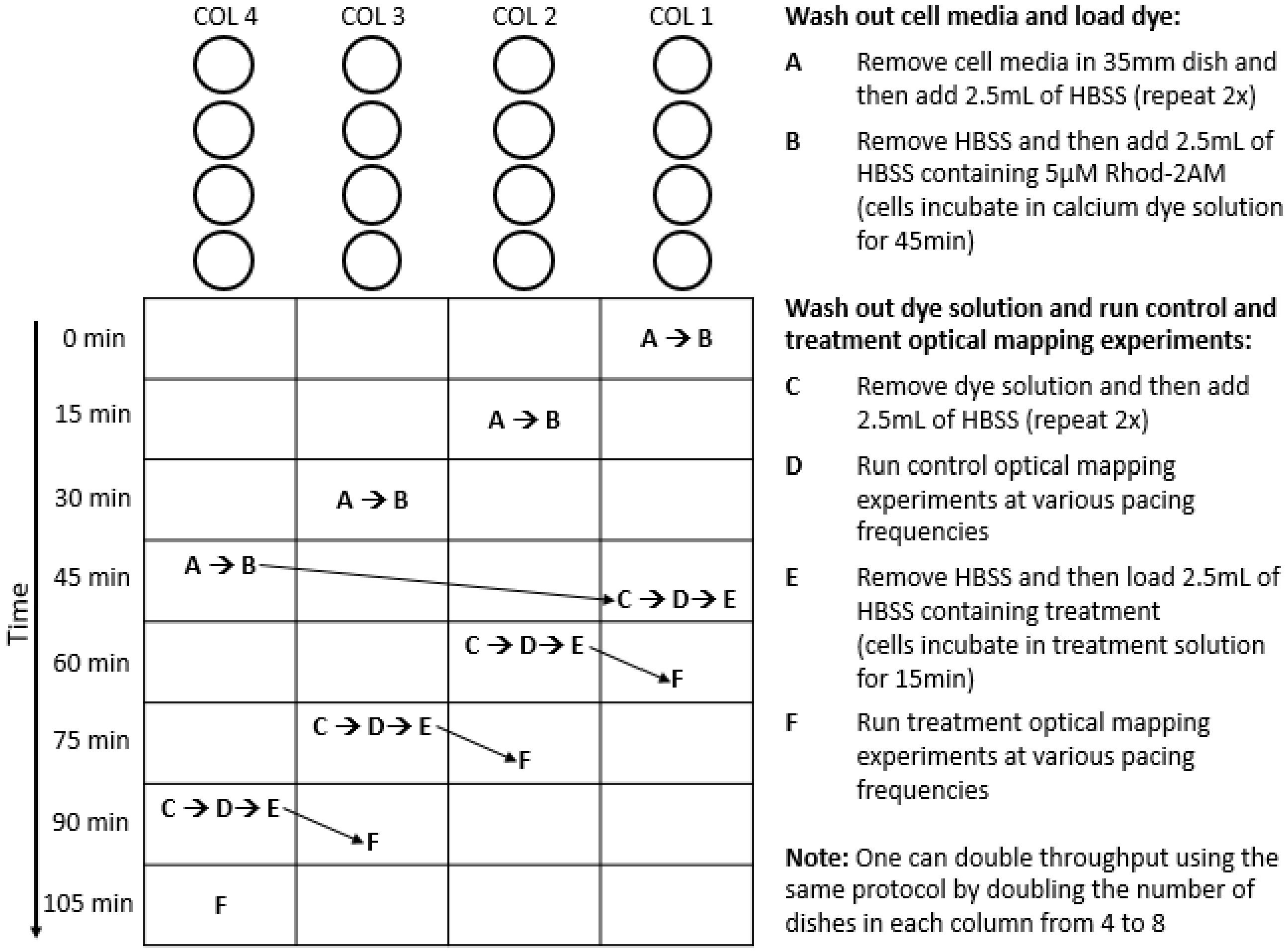

Suppl. Figure 2. Example protocol.
An outline of the experimental protocol followed by the robot. The tasks were pipelined because of the dye and treatment incubation times and the time needed to perform fluid-handling and optical mapping tasks.
A →B: Execute tasks A and B, in that order; **C → D → E:** Execute tasks C, D and E, in that order; **F:** Execute task F.

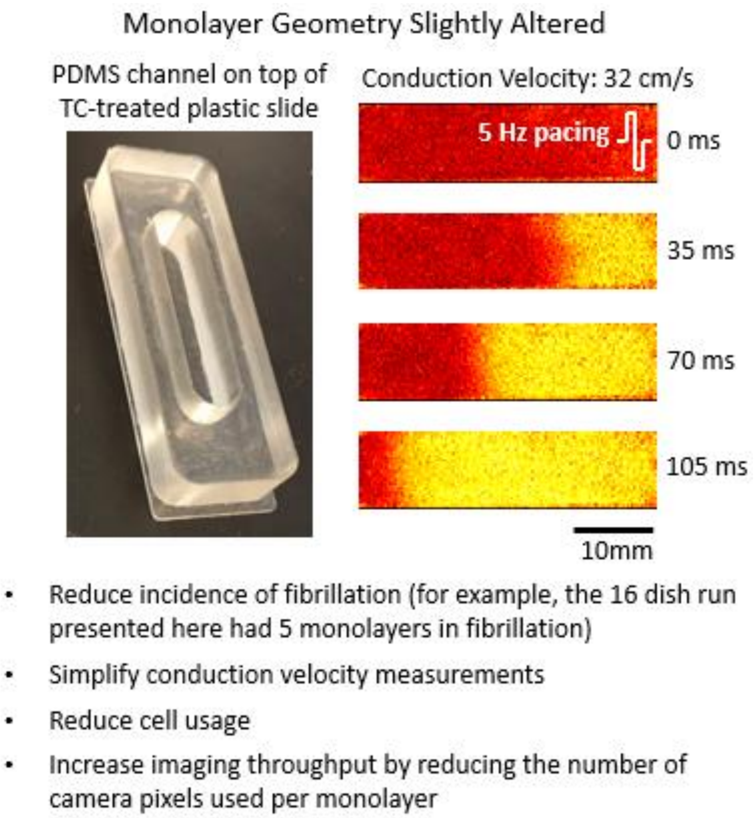

Suppl. Figure 3. Altering the monolayer geometry to minimize the incidence of fibrillation. Normalized fluorescence intensity maps at progressive time points of a monolayer formed in a custom chamber during 5Hz electrical pacing. The stimulation site is shown at the right side of the chamber (top panel). The chamber was made by attaching a PDMS channel on top of a tissue-culture-treated polystyrene slide using a silicone glue. This small-width rectangular shape drastically reduced the incidence of fibrillation (data not shown) and simplified conduction velocity measurements.

Suppl. Table 1. Optical Mapping Robot Components and Cost

Perfusion system			
**Component**	**Part #**	**Supplier**	**Cost**
Four 6-port syringe pumps<br>(to add precise amounts of liquid into 35mm dishes)	Pump XLP6K 6+ 1/4-28<br>(qnty: 4)<br><br>Syringe XLP 10.0ML<br>(qnty: 4)	Tecan US Inc.<br>Morrisville, NC, USA	$5,170
Four low-flow peristaltic pumps<br>(to remove liquid from 35mm dishes)	T60&WX10 Series<br>OEM Peristaltic Pump<br>(qnty: 4)	Longer Precision Pump Co. Ltd.<br>Baoding City, Hebei Province, China<br><br>US distributor:<br>Langer Instruments Corp<br>Tucson, AZ, USA	$430
Two high-flow peristaltic pumps<br>(to add and remove liquid from the ultrasonic bath)	Kamoer KPHM600<br>(12VDC, 600 mL/min)<br>(qnty: 2)	Amazon.com<br>ASIN: B09MVPJXFJ	$80
			**Sub-total (rounded to the nearest $10): ~ $5,680**
**2-axis XZ Cartesian robot (with perfusion and stimulation heads)**			
X-axis stage	FSL40 Ball Screw<br>Linear Motion Guide<br>(300mm stroke)<br>(qnty: 1)	Fuyu Technology Co. Ltd.<br>Chengdu city, Sichuan province, China<br><br>US distributor:<br>Amazon.com<br>ASIN: B077QLVRRB	$170
X-axis linear rail for extra mechanical support	HGH15CAZ0 (Hiwin HG 15mm Bearing Block, Square)<br>(qnty: 1)<br><br>HGR15R (Hiwin HG 15mm Linear Rail, 400mm Length)<br>(qnty: 1)	Braas Company<br>Chicago, IL, USA	$100
Z-axis stage	FSL40 Ball Screw<br>Linear Motion Guide<br>(100mm stroke)<br>(qnty: 1)	Fuyu Technology Co. Ltd.<br>Chengdu city, Sichuan province, China<br><br>US distributor:<br>Amazon.com<br>ASIN: B0784G4TYM	$150
Two stepper motor drivers	G251X (qnty: 2)	Geckodrive Inc.<br>Santa Ana, CA, USA	$140
Two optical position sensors	OPB916BZ (qnty: 2)	Mouser Electronics<br>Mansfield, TX, USA	$10
Eight graphite electrodes	POCO EDM-3 Mini Rod, 0.0625” diameter, 1.25” long (qnty: 8)	Saturn Industries Inc.<br>Hudson, NY, USA	$20
Stainless-steel inlet/outlet tubing	316 Stainless Steel Tubing, 0.062" OD, 0.005" Wall Thickness (89875K23)	McMaster-Carr Supply Company<br>Elmhurst, IL, USA	$20

	(qnty: 1 ft length)		
**Sub-total (rounded to the nearest $10): ~ $610**			
**Ultrasonic bath**			
Bath with two attached piezo-electric transducers, including transducer drive electronics which can be controlled with a relay	iSonic DS300<br>(qnty: 1)	iSonic Inc.<br>Chicago, IL, USA	$60
**Sub-total (rounded to the nearest $10): ~ $60**			
**Aluminum base-plate temperature control system**			
Temperature controller, Pt100 RTD sensor and relay	SYL-2362 (qnty: 1)<br><br>RTD-AUTO2 (qnty: 1)<br><br>SRDD100 (qnty: 1)	Auber Instruments Inc.<br>Alpharetta, GA, USA	$90
Two 72 W resistive heaters and power supply	HS150 8R F (qnty: 2)<br><br>LRS-150-24 (qnty: 1)	Mouser Electronics<br>Mansfield, TX, USA	$50
**Sub-total (rounded to the nearest $10): ~ $140**			
**Air temperature control system**			
Temperature controller, Pt100 RTD sensor and relay	SYL-2362 (qnty: 1)<br><br>Pt100MN (qnty: 1)<br><br>SRDD100 (qnty: 1)	Auber Instruments Inc.<br>Alpharetta, GA, USA	$90
One 72 W resistive heater and power supply	HS150 8R F (qnty: 1)<br><br>LRS-150-24 (qnty: 1)	Mouser Electronics<br>Mansfield, TX, USA	$40
**Sub-total (rounded to the nearest $10): ~ $130**			
**1-axis X Cartesian robot (imaging head)**			
X-axis stage	FSL40 Ball Screw Linear Motion Guide (300mm stroke)<br>(qnty: 1)	Fuyu Technology Co. Ltd.<br>Chengdu city, Sichuan province, China<br><br>US distributor:<br>Amazon.com<br>ASIN: B077QLVRRB	$170
X-axis linear rail for extra mechanical support	HGH15CAZ0 (Hiwin HG 15mm Bearing Block, Square)<br>(qnty: 1)<br><br>HGR15R (Hiwin HG 15mm Linear Rail, 400mm Length)<br>(qnty: 1)	Braas Company<br>Chicago, IL, USA	$100
Stepper motor driver	G251X (qnty: 1)	Geckodrive Inc.<br>Santa Ana, CA, USA	$70
Optical position sensor	OPB916BZ (qnty: 1)	Mouser Electronics<br>Mansfield, TX, USA	$10
**Sub-total (rounded to the nearest $10): ~ $350**			
**Option 1: Imaging head (oblique excitation)**			
Four high-speed CMOS cameras	IDS UI-3060CP2-M-GL<br>(qnty: 4)	1stVision Inc.<br>Andover, MA, USA	$4,140
Four high-speed camera lenses	Fujinon DF6HA-1S<br>(qnty: 4)	RMA Electronics Inc.<br>Hingham, MA, USA	$480

Four emission filters	ET525/50m<br>(qnty: 4)	Chroma Technology Corp<br>Bellows Falls, VT, USA	$1,300
Four excitation filters	AT480/30x<br>(qnty: 4)	Chroma Technology Corp<br>Bellows Falls, VT, USA	$800
Four collimating lenses	LA1951<br>(qnty: 4)	Thorlabs Inc.<br>Newton, NJ, USA	$110
Four high-power LEDs	LE B P1MQ-FPFT-23-0<br>(qnty: 4)	Mouser Electronics<br>Mansfield, TX, USA	$80
			**Sub-total (rounded to the nearest $10): ~ $6,910**
**Option 2: Imaging head (perpendicular excitation)**			
Four high-speed CMOS cameras	IDS UI-3060CP2-M-GL<br>(qnty: 4)	1stVision Inc.<br>Andover, MA, USA	$4,140
Four high-speed camera lenses	DO-1795<br>(qnty: 4)	Navitar Inc.<br>Rochester, NY, USA	$2,220
Four emission filters	ET590/50m<br>(qnty: 4)	Chroma Technology Corp<br>Bellows Falls, VT, USA	$1,300
Four dichroic mirrors	T560LPXR<br>(qnty: 4)	Chroma Technology Corp<br>Bellows Falls, VT, USA	$900
Four excitation filters	FF01-534/20-25<br>(qnty: 4)	Semrock Inc.<br>Rochester, NY, USA	$1,520
Four sets of optomechanical components to hold the optics and four collimating lenses	C6WR (qnty: 4)<br><br>FFM1 (qnty: 4)<br><br>B3C (qnty: 4)<br><br>LA1951 (qnty: 4)	Thorlabs Inc.<br>Newton, NJ, USA	$740
Four high-power LEDs	CBT-90-G-L11-CM101<br>(qnty: 4)	Mouser Electronics<br>Mansfield, TX, USA	$300
			**Sub-total (rounded to the nearest $10): ~ $11,120**
**Miscellaneous**			
• Raw materials: aluminum, polycarbonate plastic and acetal plastic (mechanical design and machining performed in-house)<br>• Custom electronics: LED driver, electric stimulator, etc. (electronics design and prototyping performed in-house)		Raw materials were acquired from local suppliers:<br><br>Mississauga Steel Mart<br>Mississauga, Ontario, Canada<br><br>Johnston Industrial Plastics Ltd<br>Toronto, Ontario, Canada<br><br>All electronic components, including the LEDs, were acquired from major electronic components distributors:<br><br>Digi-Key Electronics<br>Thief River Falls, MN, USA<br><br>Mouser Electronics<br>Mansfield, TX, USA	$1,000
			**Sub-total (rounded up to the nearest $1,000): ~ $1,000**
			**Total cost of robot system components: ~ $14,880 USD (Option 1)<br>~ $19,090 USD (Option 2)**
